# Retraction Note: A vehicular network based intelligent transport system for smart cities using machine learning algorithms

**DOI:** 10.1038/s41598-024-78141-8

**Published:** 2024-11-06

**Authors:** J. Prakash, L. Murali, N. Manikandan, N. Nagaprasad, Krishnaraj Ramaswamy

**Affiliations:** 1 Computer Science and Engineering, Hindusthan College of Engineering and Technology, Coimbatore, Tamil Nadu India; 2 Department of Electronics and Communication Engineering, P. A. College of Engineering, Pollachi, Coimbatore, Tamil Nadu 642 002 India; 3 Department of Mechanical Engineering, P. A. College of Engineering and Technology, Pollachi, Tamil Nadu 642002 India; 4 Department of Mechanical Engineering, ULTRA College of Engineering and Technology, Madurai, Tamil Nadu 625104 India; 5 Centre for Excellence-Indigenous Knowledge, Innovative Technology Transfer and Entrepreneurship, Dambi Dollo University, Dambi Dolo, Ethiopia; 6 Department of Mechanical Engineering, College of Engineering and Technology, Dambi Dollo University, Dambi Dolo, Ethiopia

Retraction of: *Scientific Reports* 10.1038/s41598-023-50906-7, published online 03 January 2024.

The Editors have retracted this Article because it appears to overlap significantly with a previously published article by different authors^1^. Additionally, Fig. 1 appears to be highly similar to Fig. 1 in Ref.^2^ without proper attribution.

Authors J. Prakash, M. Manikandan, N. Nagaprasad and Krishnaray Ramaswamy agree with this retraction. Author L. Murali has not responded to correspondence regarding this retraction.
